# Lymph node ratio may predict the benefit of postoperative radiotherapy in node-positive cervical cancer

**DOI:** 10.18632/oncotarget.8840

**Published:** 2016-04-18

**Authors:** Juan Zhou, Qiong-Hua Chen, San-Gang Wu, Zhen-Yu He, Jia-Yuan Sun, Feng-Yan Li, Huan-Xin Lin, Ke-Li You

**Affiliations:** ^1^ Department of Obstetrics and Gynecology, The First Affiliated Hospital of Xiamen University, Xiamen, People's Republic of China; ^2^ Department of Radiation Oncology, The First Affiliated Hospital of Xiamen University, Xiamen, People's Republic of China; ^3^ Department of Radiation Oncology, Sun Yat-sen University Cancer Center, State Key Laboratory of Oncology in South China, Collaborative Innovation Center of Cancer Medicine, Guangzhou, People's Republic of China; ^4^ Department of Gynecology, GuangDong General Hospital, Guangzhou, People's Republic of China

**Keywords:** cervical cancer, lymph node ratio, postoperative radiotherapy, survival, SEER

## Abstract

The standard treatment for node-positive cervical cancer after radical hysterectomy is pelvic radiotherapy and concurrent chemotherapy. Given the potential toxicity of postoperative radiotherapy, we used the lymph node ratio (LNR) to assess the benefit of postoperative radiotherapy in lymph node-positive cervical cancer patients. Data from the Surveillance Epidemiology and End Results database (1988–2010) were analyzed using Kaplan–Meier and Cox regression proportional hazard analysis. A total of 2,269 eligible patients were identified (median follow-up, 78.0 months); 1,863 (82.1%) patients received postoperative radiotherapy. In both univariate and multivariate analysis multivariate analysis, a higher LNR was significantly associated with a poorer outcome. A LNR > 0.16 was associated with poorer cervical cancer-related survival (CCSS) (hazard Ratio [HR] 1.376, confidence interval [CI] 1.082–1.750; P < 0.001) and overall survival (OS) (HR 1.287, CI 1.056–1.569; *P* = 0.012). Postoperative radiotherapy was only associated with survival benefits in patients with a LNR > 0.16 (CCSS, *P* < 0.001; OS, *P* < 0.001) and not in patients with a LNR ≤ 0.16 (CCSS, *P* = 0.620; OS, *P* = 0.167); these trends were not affected by number of removed lymph nodes. A higher LNR is associated with a poorer survival in lymph node-positive cervical cancer. The survival benefits of postoperative radiotherapy appear to be limited to patients with a LNR > 0.16.

## INTRODUCTION

Uterine cervical cancer will be responsible for an estimated 12,900 new cases and 4,100 deaths in the United States in 2015 [1]. Radical hysterectomy and pelvis with or without para-aortic lymphadenectomy is a standard treatment for early-stage cervical cancer. Although lymph node status is a prognostic factor, the current International Federation of Gynecology and Obstetrics (FIGO) staging system does not assess this feature [2]. The 7th edition of the American Joint Committee on Cancer (AJCC)/Union for International Cancer Control (UICC) staging system only classifies the lymph nodes as N0 (negative) or N1 (positive) [3].

However, the lymph node ratio (LNR), the ratio between the number of positive lymph nodes and removed lymph nodes (RLNs), is an important prognostic factor in breast, esophageal colorectal and other cancers, and has recently been reported to have prognostic value of survival in cervical cancer [4–18].

The LNR accurately reflects the patient's lymph node status and may enable selection of the optimal treatment. Postoperative radiotherapy (RT) is prescribed for patients with known risk factors, including positive lymph nodes, parametrical invasion, bulky tumors and a positive resection margin [19]. However, severe RT-induced toxicity on the gastrointestinal system, bones and sexual function negatively affect patient quality of life [20]. Therefore, it would be desirable to be able to identify subgroups of patients with cervical cancer who could be spared RT. The LNR has predictive significance for the benefit of postoperative RT in oral cavity cancer [21]. Thus, in this study, we used the data from the Surveillance Epidemiology and End Results (SEER) database to investigate the predictive value of the LNR for the benefit of postoperative RT in lymph node-positive cervical cancer.

## RESULTS

### Demographic and clinicopathological characteristics

In total, 2,269 eligible patients were identified (Table 1). Median age of diagnosis was 43 years (range, 18–97 years); 66.9% (1518/2269) of patients had squamous cell carcinoma; 71.7% (1627/2269) and 28.3% (642/2269) of patients had FIGO stage I and II uterine cervical cancer, respectively.

**Table 1:** Clinicopathologic characteristics of the cervical cancer patients with lymph node-positive cervical cancer stratified by postoperative radiotherapy

Variable	*N*	No RT (%)	RT (%)	*P* value
Year of diagnosis				
1988–1992	212	53 (13.1)	159 (8.5)	0.020
1993–1997	329	62 (15.3)	267 (14.3)	
1998–2002	575	89 (21.9)	486 (26.1)	
2003–2010	1153	202 (49.8)	951 (51.0)	
Race				
Black	206	49 (12.1)	157 (8.4)	0.026
White	1798	303 (74.6)	1495 (80.2)	
Other	265	54(13.2)	211 (11.3)	
Age (years)				
< 50	1559	266 (65.5)	1293 (69.4)	0.126
≥ 50	710	140 (34.5)	570 (30.6)	
Tumor histology				
Squamous	1518	271 (66.7)	1247 (66.9)	0.807
Adenocarcinoma	469	81 (20.0)	388 (20.8)	
Other	282	54 (13.3)	228 (12.2)	
Grade (*n* = 2,072)				
Well-differentiated	100	16 (4.5)	84 (4.9)	0.895
Moderately-differentiated	811	137 (38.5)	674 (39.3)	
Poorly/undifferentiated	1161	203 (57.0)	958 (55.8)	
FIGO stage				
I	1627	300 (73.9)	1327 (71.2)	0.280
II	642	106 (26.1)	536 (28.8)	
LNR				
0.01–0.16	1576	287 (70.7)	1289 (69.2)	0.552
> 0.16	693	119 (29.3)	574 (30.8)	

Abbreviations: RT, radiotherapy; FIGO, International Federation of Gynecology and Obstetrics; LNR, lymph node ratio.

**Figure 1:**
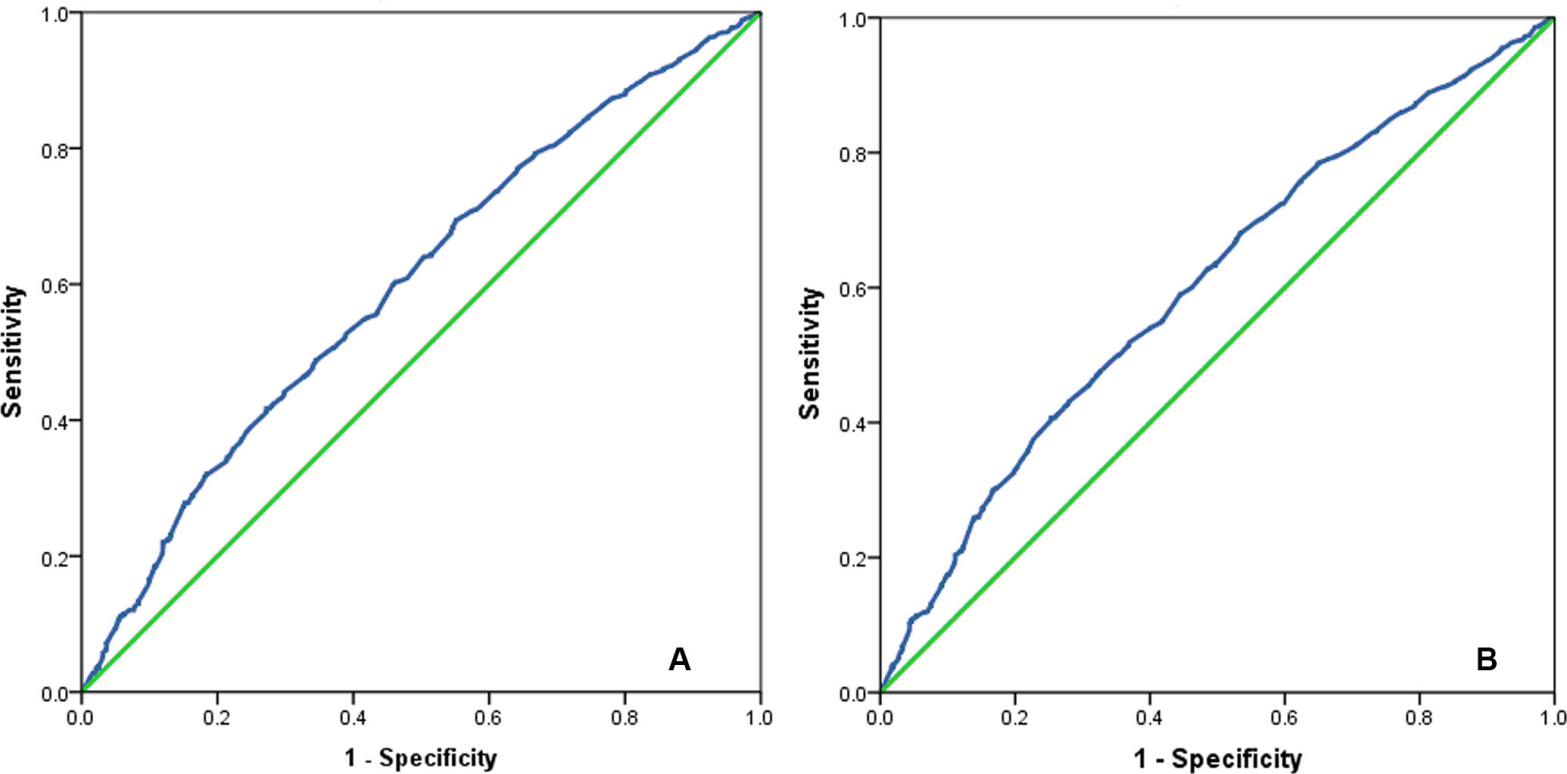
The receiver operating characteristic curve for predicting cervical cancer-related survival (A) and overall survival (B) of cervical cancer patients using lymph node ratio

The median number of RLNs was 19 (range, 1–88), the median number of positive lymph nodes was 2 (range, 1–32), and the median LNR was 0.09 (range, 0.01–1.00). An LNR of 0.16 was identified as the optimal cut-off point (cervical cancer-related survival [CCSS], Area Under receiver operating characteristic (ROC) curve = 0.599, *P* < 0.001; overall survival [OS], Area Under ROC curve = 0.602, *P* < 0.001) and used to assess the prognostic and predictive value of the LNR(Figure 1A–1B).

A total of 1,863 (82.1%) patients received postoperative RT. Patients who received postoperative RT were more likely to be White and more likely to be diagnosed between 1998 and 2002. Age, tumor histology, grade, FIGO stage and LNR were not significantly associated with postoperative RT (Table 1).

### Survival outcomes and prognostic analysis

Median follow-up for all patients was 78.0 months (range, 1–298). A total of 794 (35.0%) patients died during follow-up; 67.9% (539/794) died of cervical cancer-related disease. The 5- and 10-year CCSS rates were 77.9% and 74.0%, respectively; 5- and 10-year OS were 71.4% and 64.4%, respectively.

In both univariate and multivariate analysis, year of diagnosis, tumor histology, grade, FIGO stage, the number of positive lymph nodes, LNR and postoperative RT were significantly associated with CCSS (all *P* < 0.05). Age at diagnosis and the number of RLNs were significantly associated with CCSS in the univariate analysis, but not in multivariate analysis. Year of diagnosis, age at diagnosis, tumor histology, FIGO stage, the number of positive lymph nodes, the number of RNLs, LNR and postoperative RT were associated with OS in univariate and multivariate analysis (all *P* < 0.05; Tables 2 and 3).

**Table 2:** Univariate Cox regression analysis of prognostic factors influencing the survival of patients with lymph node-positive cervical cancer

Variable	CCSS			OS		
	HR	95% CI	*P* value	HR	95% CI	*P* value
Year of diagnosis (continuous variable)	0.967	0.953–0.982	< 0.001	0.973	0.961–0.986	< 0.001
Race						
Black	1			1		
White	0.878	0.663–1.163	0.367	0.882	0.699–1.113	0.291
Other	0.879	0.610–1.268	0.490	0.951	0.706–1.281	0.740
Age (years)						
< 50	1			1		
≥ 50	1.289	1.079–1.540	0.005	1.803	1.565–2.077	< 0.001
Tumor histology						
Squamous	1			1		
Adenocarcinoma	1.720	1.412–2.096	< 0.001	1.5643	1.393–1.937	< 0.001
Other	1.791	1.416–2.267	< 0.001	1.632	1.339–1.990	< 0.001
Grade						
Well-differentiated	1			1		
Moderately-differentiated	1.233	0.759–2.003	0.397	1.004	0.699–1.442	0.983
Poorly/undifferentiated	1.566	0.973–2.519	0.065	1.209	0.848–1.723	0.293
FIGO stage						
I	1			1		
II	1.824	1.533–2.169	< 0.001	1.834	1.589–2.117	< 0.001
LNR						
0.01–0.12	1			1		
> 0.12	1.850	1.558–2.197	< 0.001	1.830	1.588–2.109	< 0.001
LNR (continuous variable)	3.495	2.432–5.023	< 0.001	3.434	2.540–4.644	< 0.001
Postoperative RT						
No	1			1		
Yes	0.783	0.634–0.966	0.022	0.745	0.628–0.884	0.001
Number of positive lymph nodes (continuous variable)	1.095	1.069–1.122	< 0.001	1.089	1.066–1.113	< 0.001
Number of RLNs (continuous variable)	0.991	0.985–0.998	0.015	0.988	0.983–0.994	< 0.001

Abbreviations: CCSS, cervical cancer-related survival; OS, overall survival; HR, hazard ratio; CI, confidence interval; RT, radiotherapy; FIGO, International Federation of Gynecology and Obstetrics; LNR, lymph node ratio; RLNs, removed lymph nodes.

**Table 3:** Multivariate Cox regression analysis of prognostic factors influencing the survival of patients with lymph node-positive cervical cancer

Variable	CCSS			OS		
	HR	95% CI	*P* value	HR	95% CI	*P* value
Year of diagnosis (continuous variable)	0.963	0.948–0.977	< 0.001	0.967	0.954–0.979	< 0.001
Age (categorical variable)	1.148	0.957–1.376	0.136	1.161	1.399–1.866	< 0.001
Tumor histology	1.393	1.252–1.551	< 0.001	1.340	1.225–1.467	< 0.001
FIGO stage	1.748	1.467–2.083	< 0.001	1.657	1.430–1.919	< 0.001
LNR (categorical variable)	1.376	1.082–1.750	< 0.001	1.287	1.056–1.569	0.012
Number of RLNs (continuous variable)	0.992	0.983–1.000	0.057	0.990	0.0.983–0.997	0.004
Number of positive lymph nodes (continuous variable)	1.073	1.033–1.114	< 0.001	1.071	1.399–1.866	< 0.001
RT	0.765	0.619–0.945	0.013	0.729	0.614–0.865	< 0.001

Abbreviations: CCSS, cervical cancer-related survival; OS, overall survival; HR, hazard ratio; CI, confidence interval; RT, radiotherapy; FIGO, International Federation of Gynecology and Obstetrics; LNR, lymph node ratio; RLNs, removed lymph nodes.

### LNR and outcome of postoperative RT

There were 1,576 (69.5%) and 693 (30.5%) patients with LNR ≤ 0.16 and LNR > 0.16, respectively. The LNR classifications was associated with year of diagnosis, age at diagnosis, grade, and FIGO stage (*P* > 0.05 for all) (Table 4). LNR was associated with CCSS and OS in both univariate and multivariate analyses, a higher LNR was significantly associated with a poorer outcome. This association remained true whether or not the number of positive lymph nodes and the number of RNLs were included in the multivariate analysis model. In addition, LNR was prognostic in both patients who received postoperative RT and those who did not. We were not further analysis the effect of the number of positive lymph nodes and the number of RLNs on survival given the inherent relationship between the number of positive lymph nodes, the number of RLNs and LNR.

**Table 4:** Correlation between lymph node ratio classifications and clinicopathological characteristics of patients with lymph node-positive cervical cancer

Variable	LNR ≤ 0.16 (%)	LNR > 0.16 (%)	*P* value
Year of diagnosis			
1988–1992	161 (10.2)	51 (7.4)	0.044
1993–1997	235 (14.9)	94 (13.6)	
1998–2002	406 (25.8)	169 (24.4)	
2003–2010	774 (49.1)	379 (54.7)	
Race			
Black	137 (8.7)	69 (10.0)	0.540
White	1258 (79.8)	540 (77.9)	
Other	181 (11.5)	84 (12.1)	
Age (years)			
< 50	1125 (71.4)	434 (62.6)	< 0.001
≥ 50	451 (28.6)	259 (37.4)	
Tumor histology			
Squamous	1061 (67.3)	457 (65.9)	0.629
Adenocarcinoma	326 (20.7)	143 (20.6)	
Other	189 (12.0)	93 (13.4)	
Grade (*n* = 2,072)			
Well-differentiated	74 (5.1)	26 (4.1)	0.010
Moderately-differentiated	591 (41.0)	220 (34.9)	
Poorly/undifferentiated	776 (53.9)	385 (61.0)	
FIGO stage			
I	1183 (75.1)	444 (64.1)	< 0.001
II	393 (24.9)	249 (35.9)	
RT			
No	287 (18.2)	119 (17.8)	0.552
Yes	1289 (81.2)	574 (82.8)	

Abbreviations: RT, radiotherapy; FIGO, International Federation of Gynecology and Obstetrics; LNR, lymph node ratio.

**Figure 2:**
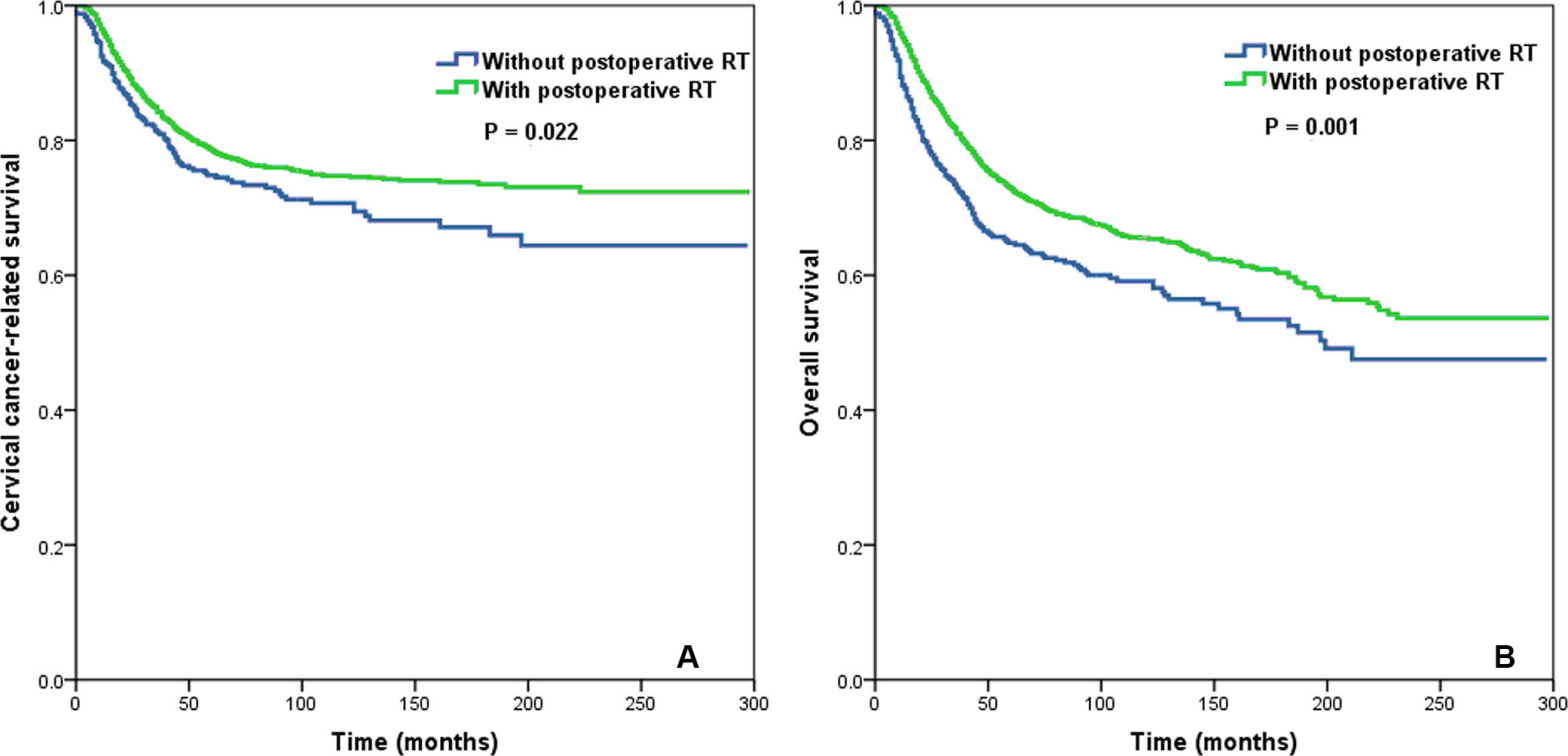
Impact of postoperative radiotherapy on cervical cancer-related survival (A) and overall survival (B) in the entire cohort of cervical cancer patients

**Figure 3:**
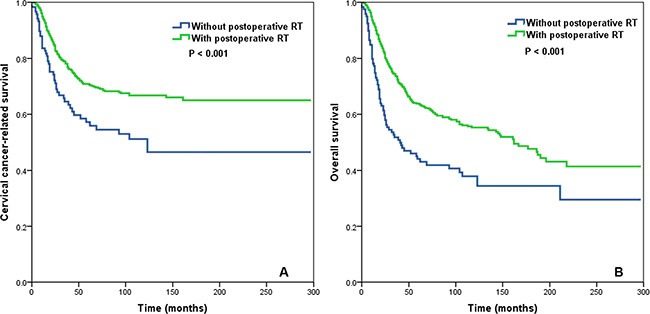
Impact of postoperative radiotherapy on cervical cancer-related survival (A) and overall survival (B) in the group of cervical cancer patients with a lymph node ratio > 0.16

In the entire cohort, postoperative RT was associated with an improvement in CCSS (*P* = 0.022) and OS (*P* = 0.001; Figure 2A–2B). Postoperative RT was associated with significantly improved CCSS (*P* < 0.001) and OS (*P* < 0.001) in patients with a LNR > 0.16 (Figure 3A–3B). Conversely, postoperative RT was not associated with CCSS (*P* = 0.620) or OS (*P* = 0.167) in patients with a LNR ≤ 0.16.

**Figure 4:**
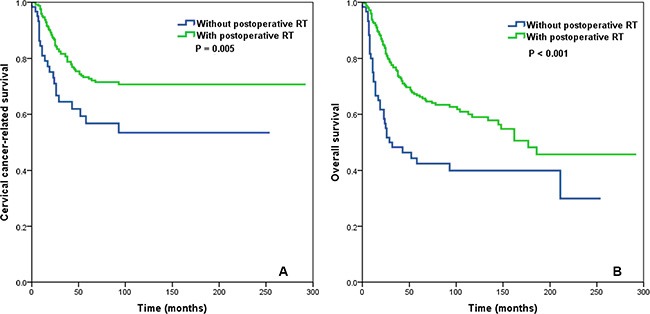
Impact of postoperative radiotherapy on cervical cancer-related survival (A) and overall survival (B) in the subgroup of cervical cancer patients with ≤ 10 removed lymph nodes and a lymph node ratio > 0.16

In patients with ≤ 10 RLNs (*n* = 422), postoperative RT was not associated with CCSS (*P* = 0.620) or OS (*P* = 0.426) in patients with a LNR ≤ 0.16. In contrast, postoperative RT was associated with improved CCSS (*P* = 0.005) and OS (*P* < 0.001 in patients with ≤ 10 RLNs and a LNR > 0.16 (Figure 4A–4B).

**Figure 5:**
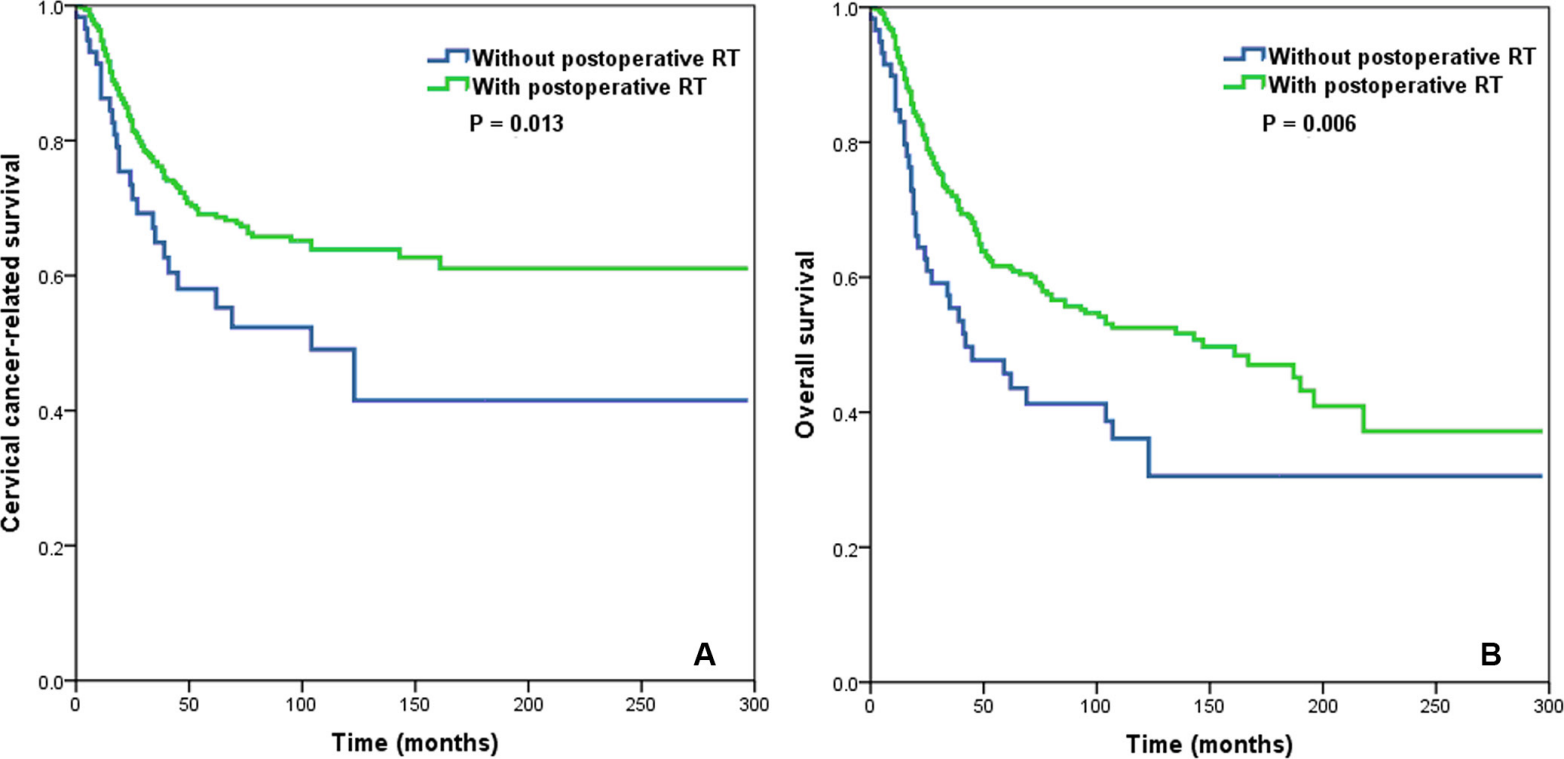
Impact of postoperative radiotherapy on cervical cancer-related survival (A) and overall survival (B) in the subgroup of cervical cancer patients with > 10 removed lymph nodes and a lymph node ratio > 0.16

In patients with > 10 RLNs (*n* = 1,847), postoperative RT was not associated with CCSS (*P* = 0.711) or OS (*P* = 0.217) in patients with a LNR ≤ 0.16. In contrast, postoperative RT was significantly improved CSSS (*P* = 0.013) and OS (*P* = 0.006) in patients with a LNR > 0.16 (Figure 5A–5B).

## DISCUSSION

The current UICC/AJCC staging system for cervical cancer does not consider the number of positive lymph nodes, though this factor significantly affects survival [22–24]. Additionally, a higher number of RLNs has a positive impact on survival in lymph node-positive cervical cancer [25]. The number of positive lymph nodes is affected by the number of RLNs; hence application of the LNR for prognostic analysis could reduce the discrepancies in assessment of lymph node status between different surgeons and pathologists [26]. Additionally, LNR could help to reduce prognostic variations due to different lymph node dissection levels [27].

The LNR been reported to have prognostic value in a range of gynecologic cancers [10–13] including cervical cancer [14–18]. Our analysis based on the SEER database demonstrates the LNR has prognostic value in lymph-node positive stage I-II cervical cancer; patients with a high LNR (> 0.16) had significantly poorer CCSS and OS.

The pelvic lymph node drainage area is the major target volume of postoperative RT in cervical cancer; RT aims to reduce local recurrence and improve survival [19]. However, no study has confirmed the value of postoperative RT for patients with different nodal disease burdens. In oral cancer, postoperative RT did not provide a survival advantage in patients with a lower LNR, but RT was significantly improved survival in patients with a higher LNR [21]. This study demonstrates postoperative RT did not affect survival in patients with a LNR ≤ 0.16, but significantly improved CCSS and OS for patients with a LNR > 0.16, and these relationships were not affected by the number of RLNs. Therefore, we recommend the LNR should be considered when prescribing postoperative RT in node-positive cervical cancer.

When compared to lymph nodes at distant anatomical locations, elevated numbers of regulatory T cells (Treg) and a decreased CD8+ T cell/Treg ratio were reported for both of the positive and negative lymph nodes in the regional lymph node area of patients with cervical cancer, which may reflect an immune suppressive microenvironment that promotes metastatic spread [28]. It is possible that patients with a higher LNR have more number of positive lymph nodes and fewer dissected lymph nodes which may indicate the presence of an immune suppressive microenvironment that could increase the risk of treatment failure; this may explain the survival benefit of postoperative RT in patients with a high LNR.

This work has several limitations. Firstly, inherent biases exist in retrospective studies. Secondly, information on tumor factors (parametrical invasion, lymphovascular invasion, margins) and treatment factors (preoperative and postoperative chemotherapy, radiotherapy techniques) could not be obtained from the SEER database. Additionally, the lack of postoperative local control and subsequent pelvic recurrence may potentially impact the clinical value of the LNR. In addition, we also found that the cut-off point of the LNR was differernt from that in previous sutdied (range: 0.066–0.10) [14–18]. However, this study is the largest analysis of the prognostic value of the LNR in cervical cancer, which reduces the potential for selection and surveillance biases.

In conclusion, the LNR is an independent prognostic factor for CCSS and OS in node-positive cervical cancer and it can be considered as a useful factor to predict the outcome of postoperative RT. Patients with a lower LNR may not benefit from postoperative RT and could avoid the associated toxicities. These findings may assist with clinical decision-making regarding postoperative RT in lymph node-positive cervical cancer; confirmation of these results in large, prospective, randomized clinical studies is warranted.

## MATERIALS AND METHODS

### Patients

Patients with a primary diagnosis of FIGO stage I–II uterine cervical cancer (International Classification of Disease for Oncology, Third Edition) between 1988 and 2010 were identified from the SEER database. Patients who received hysterectomy with pathologically-confirmed lymph node involvement were included. Patients who did not receive lymph node examinations, with an unknown number of positive lymph nodes, with unspecified/unknown radiotherapy plans, who received RT before surgery or who received radioisotope/radioactive implants were excluded. Permission was obtained to access the research data files from the SEER (reference number 11252-Nov2014) [29]. This study was approved by the ethics committees of the First Affiliated Hospital of Xiamen University.

### Demographic and clinicopathological factors

The following covariates were collected from the database: year of diagnosis, age at diagnosis, race, FIGO stage, grade, LNR and postoperative RT. Vital status, including cause of death and follow-up duration were recorded. The primary outcomes were CCSS and OS. LNR was calculated as the number of pathologically-positive lymph nodes divided by the total number of RLNs.

### Statistical analysis

Statistical analyses were performed using SPSS version 21.0 (IBM Corporation, Armonk, NY, USA). The chi-square test was used to compare demographic and clinicopathological characteristics between patients grouped by categorical variables. The optimal cut-off point for the LNR was determined by ROC curve analysis. Survival rates were calculated using the Kaplan-Meier method and compared using the log-rank test. Univariate and multivariate Cox regression analyses were performed to identify prognostic factors. Factors deemed significant in univariate analysis were included in multivariate analysis; *P* < 0.05 was considered significant in all analyses.
